# Unleashing the Power of P‐N Heterojunctions: Optimizing Water Electrolysis Efficiency Through a Cathode‐Driven Electrocatalyst Synergy

**DOI:** 10.1002/advs.75398

**Published:** 2026-04-20

**Authors:** Kumasser Kusse Kuchayita, Wei‐Nien Su, Chih‐Chia Cheng

**Affiliations:** ^1^ Graduate Institute of Applied Science and Technology National Taiwan University of Science and Technology Taipei Taiwan; ^2^ Advanced Membrane Materials Research Center National Taiwan University of Science and Technology Taipei Taiwan

**Keywords:** exfoliated molybdenum diselenide nanosheets, hydrogen evolution reaction, non‐precious metal‐based P‐N heterojunction cathode electrocatalysts, optimizing water electrolysis, oxygen evolution reaction

## Abstract

In water electrolysis, the oxygen evolution reaction (OER) at the anode requires a higher overpotential than the hydrogen evolution reaction (HER) at the cathode; thus, the activity of the anode catalyst is crucial to the overall energy consumption and reaction rate. We demonstrate an exfoliated molybdenum diselenide nanosheet (MoSe_2_)/polyaniline (PANI) system with a P‐N heterojunction that exhibits excellent HER activity as the cathode catalyst in alkaline conditions, and effectively balances the overall water electrolysis reaction and significantly enhances OER performance and anode stability. Specifically, in a dual‐electrode H‐cell, the Tafel slope of the MoSe_2_/PANI anode decreased from 115.81 mV/dec to 65.17 mV/dec, and resistance dropped from 2.5 to 0.5 Ω, indicating the MoSe_2_/PANI cathode significantly enhances the anode's catalytic ability compared to commercial platinum‐based precious metal catalysts. Importantly, this system exhibits an exceptionally low total overpotential of 1.48 V at 10 mA/cm^2^, with no significant changes in the total overpotential or Tafel slope after long‐term constant current density and cyclic voltammetry treatments. In contrast, precious metal catalyst systems degraded significantly. This groundbreaking discovery confirms that the P‐N heterojunction in the cathode catalyst is key to optimizing water electrolysis performance and provides valuable insight for future water electrolysis and emerging energy technologies.

## Graphical abstract

This groundbreaking contribution to water electrolysis demonstrates that MoSe_2_/PANI electrocatalysts with a P–N heterojunction effectively balance the overall electrolysis reaction as the cathode, enhance anode performance, and achieve high‐efficiency, low‐energy, simultaneous hydrogen and oxygen production, revealing that the P–N heterojunction enables the cathode to assist the anode and provides a synergistic effect improving overall electrolysis efficiency.

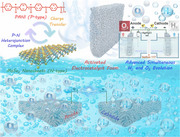

## 1 Introduction

The acceleration of the global energy transition has fueled research and the industrial development of green hydrogen, a storable, carbon‐free energy carrier. Electrochemical water splitting is widely recognized as the key technology for the sustainable production of green hydrogen [1, 2]. Despite its promising prospects, the efficiency of water electrolysis is still limited by the inherently slow kinetics of the anode oxygen evolution reaction (OER) [1, 2, 3]. The OER is a multi‐step, four‐electron, proton‐coupled oxidation process that typically requires a high overpotential to achieve the current density needed for industrial applications, and currently represents the main energy bottleneck in the entire water electrolysis process [4, 5, 6] as it leads to a significant voltage loss in practical electrolyzers, which greatly increases energy consumption and system costs [5, 7]. Therefore, the development of resource‐abundant OER electrocatalysts that offer low overpotentials and long‐term stability at high current densities remains a key challenge in the design and development of electrolyzers [7, 8].

Advanced OER catalysts based on ruthenium dioxide (RuO_2_) and iridium dioxide (IrO_2_) exhibit the highest intrinsic activity, particularly in proton exchange membrane electrolyzers. However, their large‐scale deployment is severely limited by high costs, the scarcity of these elements, and insufficient durability under harsh operating conditions, including high current densities and prolonged alkaline operation [9, 10, 11]. These limitations have driven extensive research into non‐precious metal transition metal‐based alternative catalysts. Recent benchmark tests and mechanistic studies have emphasized the inherent activity‐stability trade‐off of ruthenium/iridium oxides, which indicates the importance of rational catalyst design beyond precious metals [8, 12]. Abundant nickel (Ni), cobalt (Co), and iron (Fe)‐based catalysts have emerged as leading OER candidates for alkaline media [13, 14]. NiFe layered double hydroxides (LDHs) and their derivatives exhibit outstanding activity and durability, often comparable to or even surpassing precious metal catalysts. Through a combination of approaches, including compositional tuning, defect engineering, and hybridization with carbon or chalcogenide phases, such catalysts typically achieve overpotentials below ∼200–250 mV at a current density of 10 mA/cm^2^ and demonstrate excellent stability [15, 16]. When integrated into bifunctional structures, whether as single‐phase catalysts or heterostructures, these systems can drive overall water splitting (OWS) at cell voltages of ∼1.50–1.60 V at 10 mA/cm^2^ and sustain current densities exceeding 100 mA/cm^2^ for extended periods [17]. Recent studies further demonstrated that rational anode‐cathode pairing plays a critical role in device‐level performance, with NiFe‐based oxides, LDHs, and nitrides achieving OWS at ∼1.49–1.55 V in 1.0 m KOH—outperforming conventional platinum on carbon (Pt/C)//IrO_2_ or Pt/C//RuO_2_ systems [18, 19]. Despite these advancements, translating the electrochemical indicators of half‐cells into actual improvements in device performance remains challenging. The dual‐electrode H‐cell structure (which often includes an ion exchange membrane) is crucial for evaluating overall water splitting under practical conditions, as internal resistance, mass transport, bubble accumulation, and local pH gradients can all significantly affect performance. Numerous studies have shown that even when high‐performance OER anodes are paired with Pt/C or Ru‐based hydrogen evolution reaction (HER) cathodes, OER remains the primary source of total energy loss, with polarization effects and transport limitations often causing significant discrepancies between dual‐electrode experiments and single‐cell predictions [1, 20, 21]. These observations indicate that relying solely on high intrinsic OER activity does not guarantee efficient overall water splitting; balanced HER‐OER kinetics and efficient interfacial charge transfer are equally crucial. Therefore, the development of materials and interface engineering strategies that achieve effective power matching to bridge the gap between half‐cell performance and actual electrolyzer operation urgently needs to be explored in order to provide a promising pathway for efficient water electrolysis for hydrogen and oxygen production.

In this context, 2D transition metal dichalcogenide (TMD) materials have garnered significant attention as modular components for bifunctional electrocatalysts due to their excellent conductivity, tunable electronic structures, and abundant edge activity [22, 23]. Molybdenum diselenide (MoSe_2_) is one of the most highly regarded TMD materials due to its HER kinetics [24, 25] and, with appropriate structural tuning, promising OER and OWS performance [26]. However, although MoSe_2_ typically exhibits the desired HER performance, its inherent OER activity in a single‐cell system remains insufficient, which limits its anode efficiency when paired with high‐performance cathodes (e.g., Pt/C); thus, there is a crucial need for interface engineering strategies [27, 28]. Improving the inherent OER catalytic activity and kinetics of MoSe_2_, while overcoming the trade‐offs between durability and the complexity of synthesis, remains a significant challenge. Recent advancements in P‐N heterojunctions offer promising solutions to these challenges, as P‐N heterojunctions can enhance charge separation and facilitate interfacial charge transfer in photocatalytic and electrocatalytic systems [29, 30]. The built‐in electric field at the P‐N interface guides charge migration and regulates the adsorption energies of key OER intermediates (involving ^*^OH, ^*^O, ^*^OOH), and thereby reduces the overpotential and improves the kinetics—as demonstrated for several non‐precious metal OER catalysts [31, 32]. Extending this concept to MoSe_2_‐based systems could be highly impactful, as N‐type MoSe_2_ offers high conductivity and abundant edge or defect sites, whereas P‐type functional materials (such as conductive polymers) provide complementary band positions, redox activity, and structural flexibility [33]. This combination could facilitate the formation of P‐N heterojunctions within the matrix, enable accelerated charge transfer, stabilize the active phase under anode polarization, and improve the power balance between HER and OER, which is crucial to achieve more efficient OWS [24, 34].

We previously developed an inorganic/organic MoSe_2_/polyaniline (PANI) system with a P‐N heterojunction via electropolymerization (EP) and electroactivation (EA) that exhibited excellent pH‐universal HER catalytic performance and desirable long‐term structural stability in simulated seawater environments [35, 36]. These improvements were primarily attributed to the formation of a tightly integrated structural network between the N‐type MoSe_2_ and P‐type PANI within the composite matrix that effectively enhances structural stability and facilitates efficient charge transfer from PANI to the MoSe_2_ structure, and ultimately helps MoSe_2_ achieve high‐efficiency electrocatalytic hydrogen evolution performance. Our results also revealed that the presence of the P‐N heterojunction provides stable charge conduction pathways and modulates the reaction rate of electrocatalytic hydrogen evolution. Building on this discovery, we confidently and boldly hypothesized that, in a system with both a cathode and an anode, using MoSe_2_/PANI as the cathode catalyst has the potential to effectively adjust the overall water electrolysis reaction and enhance the catalytic performance of the anode catalyst, and thus potentially achieve simultaneous low‐energy and high‐efficiency hydrogen and oxygen production. This P‐N heterojunction strategy may enable the cathode catalyst to assist the anode catalyst and provide a synergistic enhancement that could contribute to the development of high‐efficiency, renewable energy‐driven water electrolysis.

**SCHEME 1:**
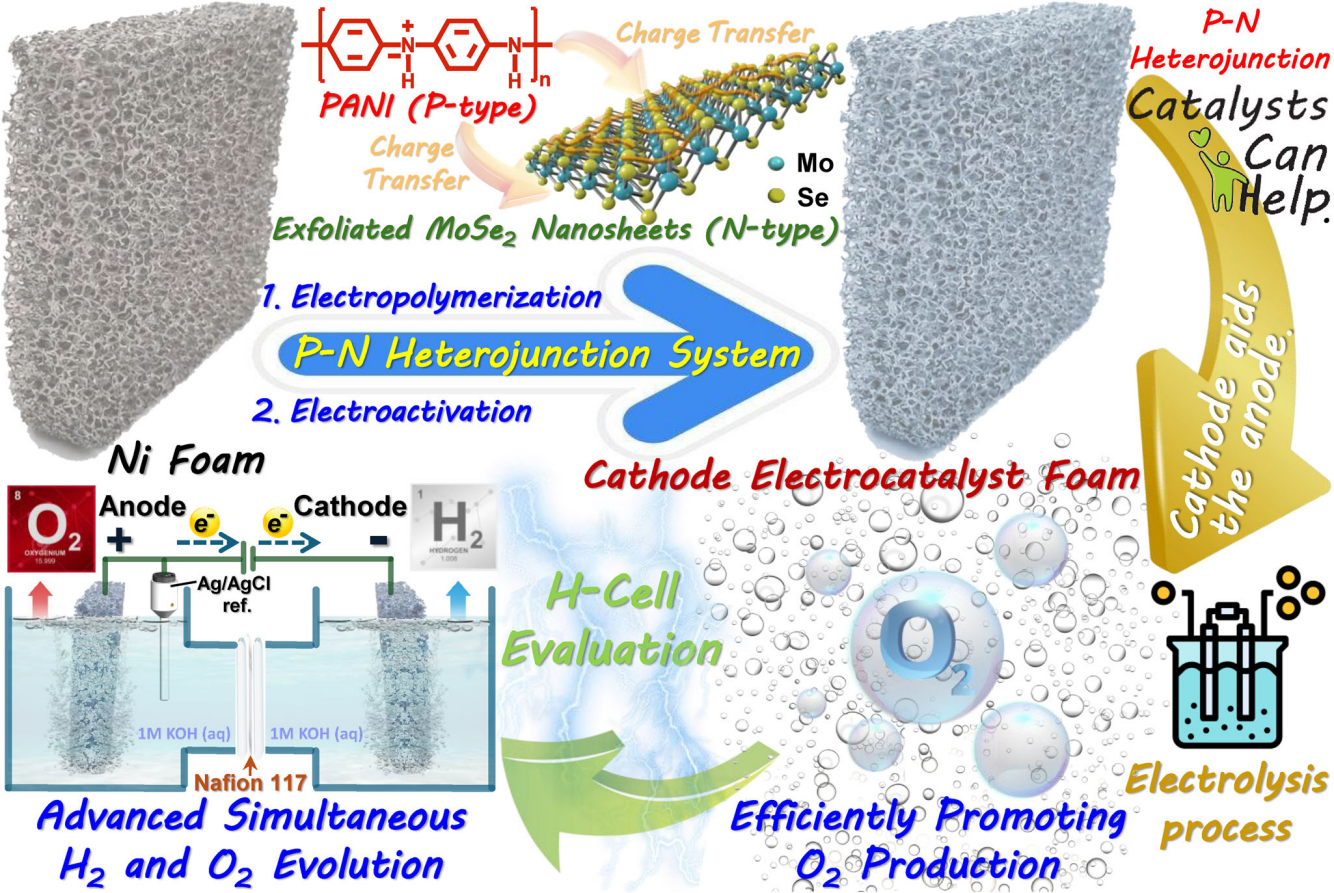
Graphical representation of the process for constructing a MoSe_2_/PANI electrocatalyst with a P‐N heterojunction on NF through electropolymerization and electroactivation and its bifunctional hydrogen and oxygen evolution capabilities. The MoSe_2_/PANI electrocatalyst significantly improves oxygen evolution catalytic ability in a single cell and, in a dual‐electrode H‐cell, enhances anode performance and balances the overall electrolysis reaction to achieve low energy consumption and high‐efficiency, simultaneous hydrogen and oxygen production.

Herein, we successfully demonstrate that a MoSe_2_/PANI electrocatalytic system, formed by combining P‐type PANI and N‐type MoSe_2_, creates a stable P‐N heterojunction structure that effectively enables the cathode to assist the catalytic ability of the anode in oxygen evolution, and thus significantly enhances overall water electrolysis performance (Scheme 1). In single‐cell testing, MoSe_2_/PANI exhibited comparable OER performance to the precious metal RuO_2_ and achieved low overpotential and long‐term stability, indicating that the P‐N heterojunction effectively enhances the oxygen evolution catalytic ability despite the limitations imposed by the theoretical potential of water splitting. Surprisingly, in dual‐electrode H‐cell testing, MoSe_2_/PANI not only maintained stable HER catalytic performance as the cathode catalyst, but also significantly enhanced the OER catalytic ability of the anode, and thereby improved overall catalytic stability. Compared to the results obtained in the single‐cell, the Tafel slope of the MoSe_2_/PANI anode in the H‐cell significantly decreased from 115.81 to 65.17 mV/dec and the resistance dropped from 2.5 Ω to approximately 0.5 Ω, clearly indicating that the MoSe_2_/PANI cathode effectively enhanced the OER catalytic performance of the MoSe_2_/PANI anode and ultimately improved oxygen evolution at the anode. More importantly, compared to the significant degradation in catalytic performance observed for the Pt/C//RuO_2_ system after long‐term stability testing, the MoSe_2_/PANI system maintained a total overpotential of only 1.48 V at 10 mA/cm^2^, with no significant changes in the total overpotential or Tafel slope of the cathode and anode, even after prolonged constant current density testing and continuous cyclic voltammetry (CV). As far as we are aware, this is the first report that the presence of a P‐N heterojunction in non‐precious metal electrocatalysts significantly enhances the overall water electrolysis reaction. This discovery holds great potential to replace commercial precious metal catalysts and improve the overall efficiency of hydrogen/oxygen production and energy conversion in water electrolysis.

## 2 Results and Discussion

**FIGURE 1:**
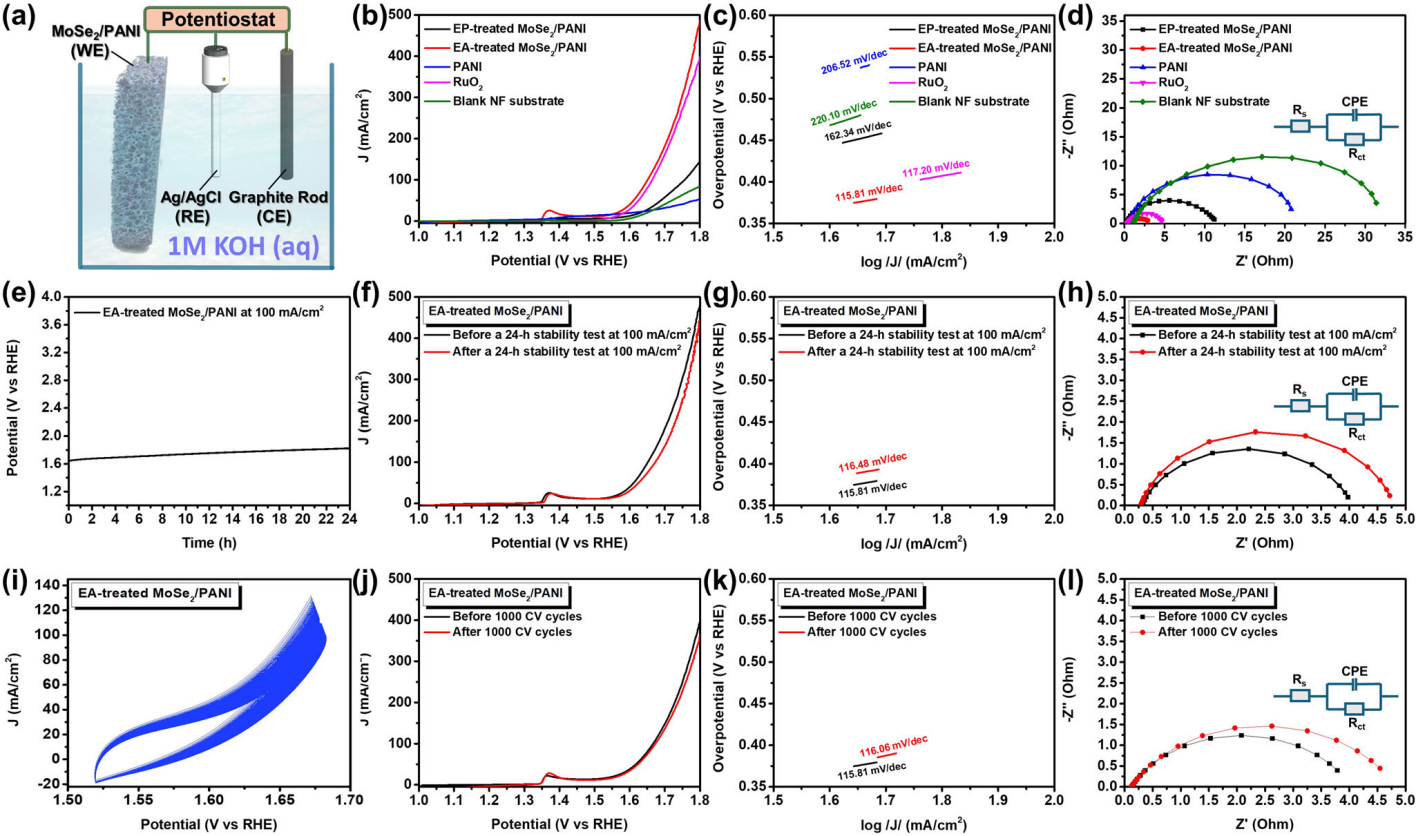
(a) Schematic of the single‐cell setup for the OER evaluation with a working electrode (WE), reference electrode (RE), and counter electrode (CE). (b) LSV curve, (c) Tafel plot, and (d) EIS spectra for MoSe_2_/PANI on NF in 1.0 m KOH solution. (e) Chronopotentiometry curve for MoSe_2_/PANI on NF during continuous operation at a fixed current density of 100 mA/cm^2^ for 24 h in 1.0 m KOH solution. Electrochemical measurements of MoSe_2_/PANI on NF in the single cell before and after 24 h of treatment at 100 mA/cm^2^ in 1.0 m KOH solution: (f) LSV curve, (g) Tafel plot, and (h) EIS spectra. (i) CV curve for MoSe_2_/PANI on NF in 1.0 m KOH solution after 1000 continuous cycles at a scan rate of 100 mV/s. Electrochemical measurements of MoSe_2_/PANI on NF in the single cell before and after 1000 cycles of CV in 1.0 m KOH solution: (j) LSV curve, (k) Tafel plot, and (l) EIS spectra. Insets in (d), (h), and (l) on the right show the equivalent circuit model used to fit the EIS data.

Based on our previous development and foundation of the inorganic‐organic MoSe_2_/PANI electrocatalyst for efficient hydrogen production through P‐N heterojunctions [35, 36], in this study, we further evaluated the impact of this P‐N heterojunction electrocatalytic system in overall water electrolysis. Specifically, we focused on assessing catalytic OER ability and whether this system effectively aids the anode electrode when acting as the cathode electrocatalyst to enhance overall water electrolysis performance (Scheme 1). The preparation of the MoSe_2_/PANI electrode followed the procedure reported in our previous work [35, 36]. MoSe_2_ was exfoliated in an aqueous solution, and EP and EA of MoSe_2_/PANI were performed on NF, ultimately resulting in the construction of the EA‐treated MoSe_2_/PANI electrode on NF. First, we evaluated the OER catalytic performance of the MoSe_2_/PANI electrocatalyst in 1 m KOH as the electrolyte to confirm whether the presence of the P‐N heterojunction enhances the catalytic oxygen evolution activity of MoSe_2_. The OER evaluation was conducted using a single‐cell electrochemical setup with a three‐electrode array, as shown in Figure 1a. Commercially available OER catalyst RuO_2_ was used as the benchmark [12]. As shown in Video S1, the MoSe_2_/PANI working electrode (WE) macroscopically exhibits stable catalytic oxygen evolution characteristics, suggesting that the system with the P‐N heterojunction possesses bifunctional catalytic capabilities for both HER and OER. Electrochemical analysis (Figure 1b–d) clearly showed that, compared to the pristine PANI and EP‐treated MoSe_2_/PANI, the EA‐treated MoSe_2_/PANI exhibited a significantly lower overpotential (1.61 V at 50 mA/cm^2^ and 1.65 V at 100 mA/cm^2^, Figure 1b), Tafel slope (115.81 mV/dec, Figure 1c), and resistance (2.5 Ω, Figure 1d) for OER catalytic performance. MoSe_2_/PANI also slightly outperformed RuO_2_, which confirmed that EA‐treated MoSe_2_/PANI has potential for OER catalysis applications. The superior OER catalytic performance of EA‐treated MoSe_2_/PANI compared with EP‐treated MoSe_2_/PANI can be primarily attributed to the EA treatment, which effectively enhances the degree of P–N heterojunction formation between MoSe_2_ and PANI [36], thereby improving the overall OER activity. Although the EA‐treated MoSe_2_/PANI exhibited a relatively lower surface catalytic area in the double‐layer capacitance (*C*_dl_) analysis compared to RuO_2_ (RuO_2_: 19.8 mF/cm^2^; MoSe_2_/PANI: 13.1 mF/cm^2^, Figure S1), it still retains excellent OER catalytic performance. This suggests that the P‐N heterojunction interface between MoSe_2_ and PANI enhances charge transfer, and thereby stabilizes the catalytic sites on the surface of MoSe_2_ to enable effective OER catalysis [36]. In addition, assessments of the surface morphology by scanning electron microscopy (SEM) and energy‐dispersive X‐ray (EDX) spectroscopy (Figures S2 and S3) clearly indicated that the MoSe_2_/PANI electrode maintained a stable morphology after the OER evaluation, with all expected elements uniformly distributed on the substrate. Similar results were observed in Raman spectroscopy analysis (Figure S4), confirming that MoSe_2_/PANI possesses excellent structural stability and can consistently perform OER catalysis in an alkaline environment.

To further confirm the stability of MoSe_2_/PANI during OER catalysis in 1 m KOH, we performed continuous operation for 24 h at a fixed current density of 100 mA/cm^2^ and evaluated the catalytic and structural characteristics of MoSe_2_/PANI after 1000 cycles of CV. As shown in Figure 1e–l, under both a current density of 100 mA/cm^2^ and after 1000 cycles of CV scans, the linear sweep voltammetry (LSV) curve, Tafel slope, and resistance only changed minimally compared to the values for the original MoSe_2_/PANI, indicating that MoSe_2_/PANI exhibits excellent OER catalytic durability. In contrast, the electrochemical catalytic properties of RuO_2_ underwent significant changes after both tests (Figure S5); for example, after 24 h at 100 mA/cm^2^, the overpotential at 50 mA/cm^2^ shifted from 1.63 to 1.67 V (Figure S5b), the Tafel slope changed from 117.20 to 172.82 mV/dec (Figure S5c), and the resistance increased from 1.8 to 4.2 Ω (Figure S5d). These results confirm that the OER catalytic performance of RuO_2_ is severely impacted by the alkaline environment, which can be primarily attributed to the oxidation of RuO_2_ into soluble ruthenate ions under alkaline conditions during water electrolysis, which leads to structural dissolution and decreases overall catalytic performance [37, 38]. This comparison with RuO_2_ further revealed that the structure of MoSe_2_/PANI, particularly the P‐N heterojunction interface, plays a crucial role in enhancing the overall stability and thereby facilitates stable catalytic oxygen evolution in an alkaline environment [36].

**FIGURE 2:**
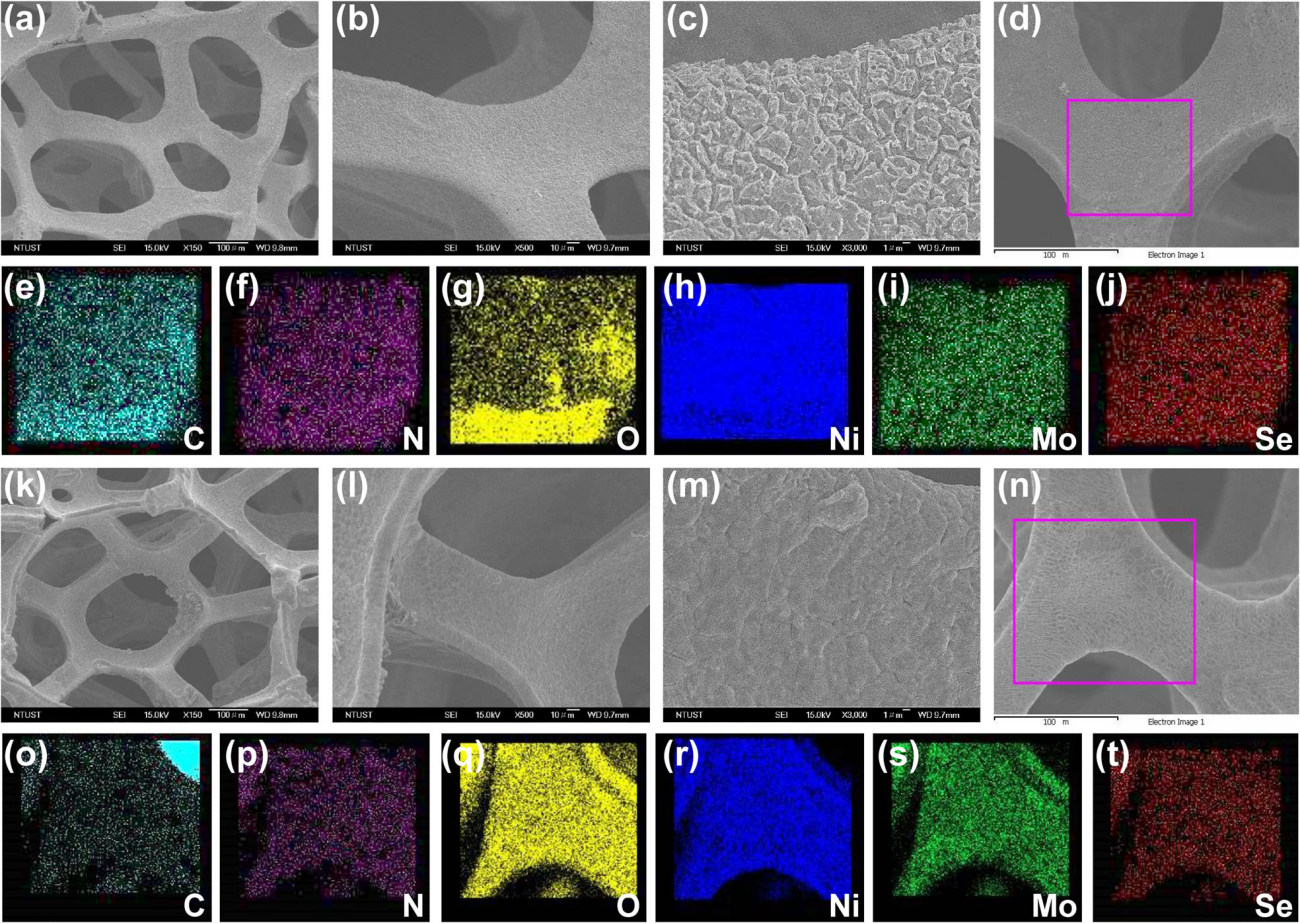
SEM images of MoSe_2_/PANI on NF after continuous operation at a fixed current density of 100 mA/cm^2^ for 24 h in 1.0 m KOH, followed by OER testing in the single cell, at magnifications of (a) ×150, (b) ×500, and (c) ×3000. (d) SEM image of MoSe_2_/PANI on NF after OER testing, used for elemental mapping analysis, with the analyzed region indicated by the purple box in the figure. Elemental mapping images corresponding to the purple box in (d): (e) C, (f) N, (g) O, (h) Ni, (i) Mo, and (j) Se. SEM images of MoSe_2_/PANI on NF after 1000 cycles at a scan rate of 100 mV/s in 1.0 m KOH, followed by OER testing in the single cell, at magnifications of (k) ×150, (l) ×500, and (m) ×3000. (n) SEM image of MoSe_2_/PANI on NF after OER testing, used for elemental mapping analysis, with the analyzed region indicated by the purple box in the figure. Elemental mapping images corresponding to the purple box in (n): (o) C, (p) N, (q) O, (r) Ni, (s) Mo, and (t) Se.

To further validate these results, we used SEM and surface element analysis to confirm the surface morphological features of MoSe_2_/PANI after 24 h operation at a current density of 100 mA/cm^2^ and after 1000 cycles of CV. As shown in Figure 2, compared to the morphology of the original MoSe_2_/PANI (Figure S2), the surface of MoSe_2_/PANI showed no significant changes after either test, with all characteristic elements still clearly distributed in the substrate, in further validation that MoSe_2_/PANI maintains stable structural integrity to support ongoing OER catalysis. Although MoSe_2_/PANI demonstrates stable OER electrocatalytic activity, many high‐performance OER catalysts, such as those involving multiple high oxidation state transition metal species on NF [39, 40], operate relatively close to the theoretical potential for water splitting (∼1.23 V vs RHE) that drives efficient OER or related oxidation reactions; this allows them to effectively overcome the slow, energy‐intensive OER kinetics [41, 42]. In other words, the OER catalytic activity of MoSe_2_/PANI may be primarily limited by the theoretical potential barrier of water splitting, which restricts its ability to fully perform and execute efficient OER oxygen evolution. Therefore, considering the overall water electrolysis reaction, when the cathode possesses an electrocatalyst with excellent HER catalytic capability that can balance the transfer of four electrons involved in the total reaction (i.e., four electrons consumed in the cathodic reduction reaction and four electrons released in the anodic oxidation reaction), MoSe_2_/PANI as the anode catalyst will have a high potential to achieve low‐energy and high‐efficiency OER performance with the assistance of the cathode catalyst.

**FIGURE 3:**
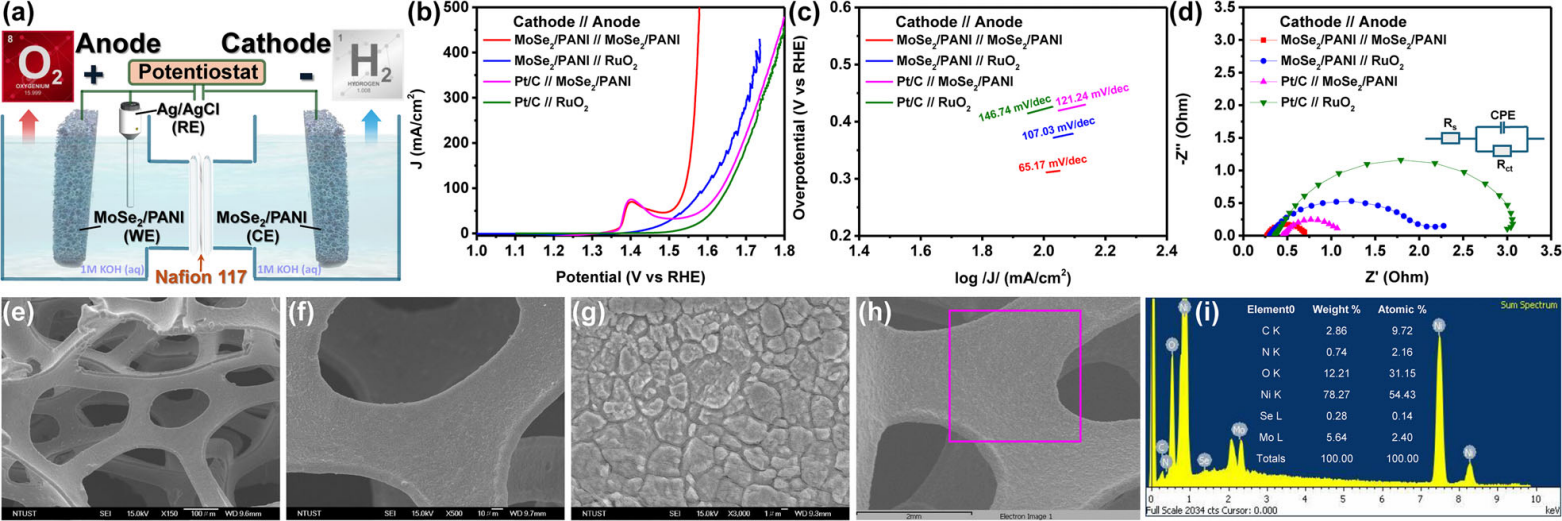
(a) Schematic of the H‐cell setup for simultaneous HER at the cathode and OER at the anode evaluation. (b) LSV curves, (c) Tafel plot, and (d) EIS spectra for MoSe_2_/PANI//MoSe_2_/PANI (cathode//anode), MoSe_2_/PANI//RuO_2_, Pt/C//MoSe_2_/PANI, and Pt/C//RuO_2_ in the H‐cell with 1.0 m KOH solution. The inset on the right side in (d) shows the equivalent circuit model used for fitting the EIS data. SEM images of MoSe_2_/PANI on NF after OER testing in the H‐cell with 1 m KOH solution, at magnifications of (e) ×150, (f) ×500, and (g) ×3000. (h) SEM image of MoSe_2_/PANI on NF after OER testing in H‐cell with 1 m KOH solution, used for EDX analysis, with the analyzed region indicated by the purple box in the figure. (i) Corresponding EDX spectrum of the region within the purple box in (h).

To confirm our hypothesis that a cathode catalyst with outstanding HER catalytic ability will assist the oxygen evolution capability of the anode catalyst, we conducted an evaluation using an H‐type electrochemical cell (H‐cell). The cathode catalyst was MoSe_2_/PANI, which already exhibits excellent pH‐universal HER electrocatalytic activity [36]; the anode was MoSe_2_/PANI, the separator membrane was Nafion 117, and the electrolyte was 1 m KOH. The cathode and anode catalyst controls were Pt/C and RuO_2_, respectively. A schematic diagram of the setup of the H‐cell is shown in Figure 3a. The LSV curves in Figure 3b show that the results for the cathode//anode combinations of Pt/C//MoSe_2_/PANI or Pt/C//RuO_2_ are nearly identical to those of the single cell (Figure 1b), indicating that both electrodes exhibit independent catalytic characteristics. Surprisingly, compared to Pt/C//MoSe_2_/PANI or Pt/C//RuO_2_, the OER catalytic ability of MoSe_2_/PANI//MoSe_2_/PANI or MoSe_2_/PANI//RuO_2_ was significantly enhanced, with noticeably reduced overpotential values at various current densities, suggesting that using MoSe_2_/PANI with a P‐N heterojunction interface as the cathode catalyst can effectively improve the OER catalytic reaction at the anode catalyst. Notably, the overpotential values for MoSe_2_/PANI//MoSe_2_/PANI at 50 and 100 mA/cm^2^ were 1.50 and 1.54 V, respectively, significantly lower than the values for MoSe_2_/PANI//RuO_2_ (1.54 and 1.59 V at 50 and 100 mA/cm^2^, respectively), suggesting that the P‐N heterojunction structure in the MoSe_2_/PANI anode catalyst effectively enhances OER catalytic activity. In other words, MoSe_2_/PANI promotes charge transfer through its own P‐N heterojunction structure at both the cathode and anode [36], which facilitates the desired synergistic effect between the electrodes. This ultimately leads to a balanced overall water electrolysis reaction and enables simultaneous high‐efficiency hydrogen and oxygen production. In addition, compared to the results obtained using the single cell (Figure 1c,d), in the H‐cell, the Tafel slope of MoSe_2_/PANI//MoSe_2_/PANI decreased significantly from 115.81 to 65.17 mV/dec (Figure 3c) and its resistance also dropped from 2.5 Ω to approximately 0.5 Ω (Figure 3d), clearly indicating that the MoSe_2_/PANI cathode effectively enhances the OER catalytic characteristics of the MoSe_2_/PANI anode, and thereby greatly improves oxygen evolution performance at the anode. These results further indicate that the MoSe_2_/PANI cathode not only balances the overall water electrolysis energy barrier but also plays a key role in enhancing the anode's catalytic performance.

The Ni in the NF substrate has high OER catalytic activity, and its characteristic signal typically appears around 1.4 V (vs RHE), which represents the redox process of Ni(II) being oxidized to Ni(III/IV), followed by oxygen evolution and generation [43]. To clarify the contributions of the NF substrate and MoSe_2_/PANI in OER catalysis, we treated MoSe_2_/PANI in a 1 m KOH environment under a constant current of 10 mA/cm^2^ for 2 h to accelerate the redox process of the NF substrate and observe the voltage changes during the process. As shown in Figure S6a, the voltage remained stable at 1.49 V during the 2 h process, indicating that MoSe_2_/PANI maintained stable structural characteristics under these conditions. Subsequently, LSV analysis (Figure S6b) revealed that, compared to the original MoSe_2_/PANI//MoSe_2_/PANI LSV curve (Figure 3b), the intensity of the oxidation peak of the NF substrate around 1.4 V in the forward LSV scan significantly decreased, indicating that the NF substrate had largely transitioned to its oxidized state after the constant current density treatment. When further backward LSV scans were performed, the oxidation peak completely disappeared, and the original parabolic LSV curve reappeared, resulting in the same corresponding overpotential values at different current densities, which confirmed that the improvement in the OER catalytic characteristics primarily originates from MoSe_2_/PANI. After conducting the H‐cell evaluation, we further explored the surface morphology and structural composition of the MoSe_2_/PANI anode catalyst using SEM and EDX. As shown in Figure 3e–i, the obtained images and elemental analysis results, which were consistent with those observed in the single cell evaluations (Figures S2 and S3), demonstrate the same surface morphology and the presence of all expected elemental features. These findings indicate that the MoSe_2_/PANI anode maintained stable structural characteristics to carry out the OER catalytic reaction during the H‐cell evaluation. These morphological results also subtly suggest that the structure of MoSe_2_/PANI is sufficiently stable to support its role as both the cathode and anode catalyst, which enables it to effectively assist the cathode in balancing the overall water electrolysis energy barrier and significantly enhance OER catalytic activity at the anode.

**FIGURE 4:**
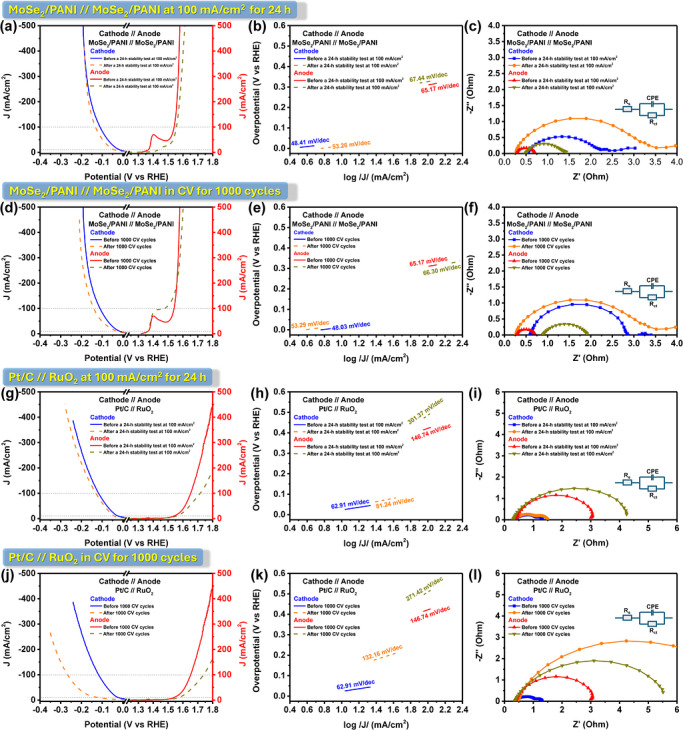
(a) Steady‐state polarization curves, (b) Tafel plot, and (c) EIS spectra for MoSe_2_/PANI//MoSe_2_/PANI (cathode//anode) on NF in the H‐cell for HER and OER before and after 24 h treatment at 100 mA/cm^2^ in 1.0 m KOH solution. (d) Steady‐state polarization curves, (e) Tafel plot, and (f) EIS spectra for MoSe_2_/PANI//MoSe_2_/PANI on NF in the H‐cell before and after 1000 cycles of CV in 1.0 m KOH solution. (g) Steady‐state polarization curves, (h) Tafel plot, and (i) EIS spectra for Pt/C//RuO_2_ on NF in the H‐cell for HER and OER before and after 24 h treatment at 100 mA/cm^2^ in 1.0 m KOH solution. (j) Steady‐state polarization curves, (k) Tafel plot, and (l) EIS spectra for Pt/C//RuO_2_ on NF in the H‐cell before and after 1000 cycles of CV in 1.0 m KOH solution. Insets in (c), (f), (i), and (l) on the right show the equivalent circuit model used to fit the EIS data.

Next, we further evaluated the catalytic and structural stability of MoSe_2_/PANI as both the cathode and anode in H‐cells by subjecting it to a 24‐h treatment at a constant current density of 100 mA/cm^2^ or continuous CV for 1000 cycles. As shown in Figure 4a–f, compared to the original values before treatment, the LSV curves and Tafel slope values for MoSe_2_/PANI//MoSe_2_/PANI at both the cathode and anode changed only slightly after either of these treatments. Specifically, before treatment, the system exhibited a very small total overpotential value of 1.65 V (between the cathode and anode at 100 mA/cm^2^, Figure 4a,d), indicating that this system possesses excellent energy efficiency and effectively enhances catalytic activity, reduces resistance, and improves mass transfer efficiency, and thereby results in outstanding simultaneous hydrogen and oxygen production performance [44, 45]. After treatment, the total overpotential of the cathode and anode increased only slightly to 1.67 V at 100 mA/cm^2^ (1.48 V at 10 mA/cm^2^, Figure 4a,d), revealing that this system possesses excellent catalytic stability, even after constant current density and CV treatment. The Tafel slopes of the cathode and anode before treatment were 48.41 and 65.17 mV/dec, respectively. After treatment, the Tafel slopes of both electrodes minimally increased to 53.26 and 67.44 mV/dec (Figure 4b,e), confirming that the MoSe_2_/PANI cathode exhibits high HER catalytic stability, which enables it to continuously maintain and support the OER catalytic activity of the MoSe_2_/PANI anode during the H‐cell measurement process, even after constant current density and CV treatments [36]. After treatment by both methods, the Tafel slopes of the cathode and anode showed only slight increases, with results consistent with the trends observed in the single cell (Figure 1g,k) and in our previous study [36], which may be attributed to the minimal passivation of the catalytic activity of both electrodes under alkaline conditions. Nevertheless, the resistance of both the cathode and anode increased significantly after the constant current density and CV treatments (Figure 4c,f). For example, in Figure 4c, after the constant current density treatment, the cathode resistance increased from 1.72 to 3.27 Ω, and the anode resistance increased from 0.41 to 0.93 Ω. This suggests that the constant current density treatment may have led to some degradation on the surfaces of both the cathode and anode, which could affect the electrode‐electrolyte interface, increase dehydration or contamination of the ion exchange membrane, and ultimately lead to a gradual increase in the ohmic losses on both sides of the electrodes [46, 47]. In other words, although MoSe_2_/PANI//MoSe_2_/PANI maintained excellent catalytic activity after the constant current density and CV treatments, the surface microstructure may have developed some defects, which would gradually impact the overall performance and long‐term reliability of the H‐cell; this is a key consideration for future extension to membrane electrode assembly (MEA) electrolyzer applications [48, 49].

In contrast to the MoSe_2_/PANI//MoSe_2_/PANI system, the Pt/C//RuO_2_ system exhibited significant degradation in the catalytic properties of both the cathode and anode after the constant current density and CV treatments (Figure 4g–l). Specifically, the total overpotential increased substantially (from the original 1.77 V at 100 mA/cm^2^ to 1.85 V after constant current density treatment and to 2.1 V after CV treatment; Figure 4g,j), the Tafel slope values changed drastically (from 62.91 and 146.74 mV/dec for the cathode and anode, respectively, to 81.24 and 301.37 mV/dec after constant current density treatment and 132.16 and 271.42 mV/dec after CV treatment; Figure 4h,k), accompanied by significant increases in resistance (Figure 4i,l). These results can be attributed to the high instability of the precious metals Pt and Ru in Pt/C and RuO_2_ in alkaline environments [37, 38, 50, 51], which leads to significant degradation of the catalytic properties of both the cathode and anode. It is noteworthy that, compared to Pt/C and RuO_2_ in the single cell (Figure S5 and our previous work [36]), the degradation of the catalytic performance of the Pt/C//RuO_2_ system in the H‐cell occurred more rapidly and significantly, indicating that catalyst deactivation and side reactions severely impact the overall electrolysis environment, the conductivity characteristics of the proton exchange membrane, and contamination effects. Therefore, these comparisons further highlight the indispensable and crucial contribution of the P‐N heterojunction structure in the MoSe_2_/PANI//MoSe_2_/PANI system to overall water electrolysis performance and long‐term stability.

To further understand the performance of the MoSe_2_/PANI//MoSe_2_/PANI system in the H‐cell, we performed a comparison with previously reported bifunctional electrocatalysts. As shown in Table S1, compared to systems with multiple elemental states or MoSe_2_ doping [33, 52, 53, 54, 55, 56, 57, 58, 59], the MoSe_2_/PANI//MoSe_2_/PANI system demonstrates the lowest total overpotential of 1.48 V at 10 mA/cm^2^, confirming that the MoSe_2_/PANI system with a P‐N heterojunction not only exhibits the expected bifunctional HER and OER electrocatalytic activity in alkaline environments, but also efficiently balances the overall water electrolysis reaction when acting as the cathode catalyst and thus enhances the oxygen evolution catalytic ability of the anode catalyst and improves the long‐term electrocatalytic stability of both electrodes. This finding is also the first time that the presence of a P‐N heterojunction in an electrocatalyst has been reported to significantly contribute to the overall water electrolysis reaction; therefore, this system holds great potential to replace commercial precious metal catalysts and also enhance the overall efficiency of hydrogen/oxygen production and energy conversion in fuel cells (or MEA).

**FIGURE 5:**
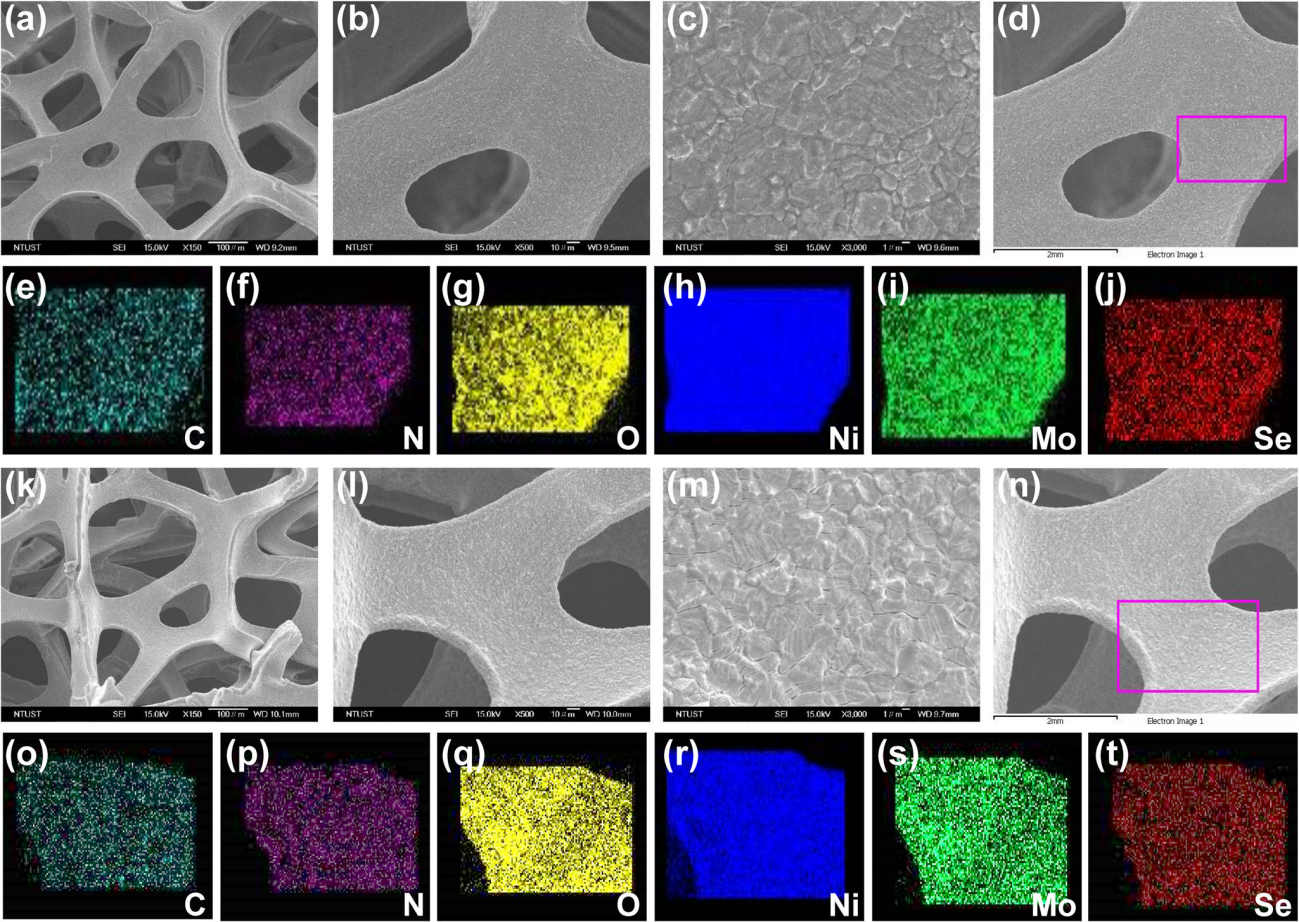
SEM images of MoSe_2_/PANI on NF after 24 h treatment at 100 mA/cm^2^ in 1.0 m KOH solution, followed by OER testing in the H‐cell, at magnifications of (a) ×150, (b) ×500, and (c) ×3000. (d) SEM image of MoSe_2_/PANI on NF after OER testing, used for elemental mapping analysis, with the analyzed region indicated by the purple box in the figure. Elemental mapping images corresponding to the purple box in (d): (e) C, (f) N, (g) O, (h) Ni, (i) Mo, and (j) Se. SEM images of MoSe_2_/PANI on NF after 1000 cycles of CV in 1.0 m KOH solution, followed by OER testing in the H‐cell, at magnifications of (k) ×150, (l) ×500, and (m) ×3000. (n) SEM image of MoSe_2_/PANI on NF after OER testing, used for elemental mapping analysis, with the analyzed region indicated by the purple box in the figure. Elemental mapping images corresponding to the purple box in (n): (o) C, (p) N, (q) O, (r) Ni, (s) Mo, and (t) Se.

Next, we performed SEM, elemental distribution, and Raman spectroscopy analysis to further confirm the structural stability of the MoSe_2_/PANI//MoSe_2_/PANI system after 24 h of constant current density at 100 mA/cm^2^ or 1000 continuous CV scans, followed by OER testing in the H‐cell. As presented in Figure 5, after treatment with both methods, the MoSe_2_/PANI//MoSe_2_/PANI system exhibited a similar morphology to the original sample (Figure S2), with all expected elements clearly observed and evenly distributed on the surface of the sample. In addition, the Raman spectroscopy data in Figure S7 show that the MoSe_2_/PANI//MoSe_2_/PANI system did not undergo any structural changes after the constant current density or CV scanning treatments. Specifically, compared to the original exfoliated MoSe_2_ nanosheets, the *A_1g_*, $E_{2g}^{1},\;\mathrm{and}$ $B_{2g}^{1}$ characteristic peaks of the MoSe_2_/PANI system remained at the same positions and had the same shapes after both treatments. These results further confirm that the system possesses excellent structural stability to support simultaneous hydrogen and oxygen production in the H‐cell. Collectively, based on these findings, the MoSe_2_/PANI electrocatalytic system with a P‐N heterojunction plays a crucial role in hydrogen production catalytic performance by effectively balancing the overall water electrolysis reaction when acting as the cathode catalyst, and thereby enhances the oxygen evolution catalytic properties of the anode catalyst, to ultimately achieve high stability and efficiency in simultaneous hydrogen and oxygen production. This emerging concept of using a cathode P‐N heterojunction electrocatalyst to enhance the catalyst performance of the anode could have a significant impact on the future development of non‐precious metal‐based electrocatalysts and fuel cells, and the advancement of various green energy technologies.

## 3 Conclusions

In summary, this study reveals the breakthrough potential of the MoSe_2_/PANI electrocatalytic system, which combines P‐type PANI and N‐type MoSe_2_ nanosheets, for water electrolysis. We demonstrate that, when used as the cathode, this catalyst significantly enhances the catalytic activity of the anode, achieving outstanding simultaneous hydrogen and oxygen production. These findings underscore the critical role of P–N heterojunctions in electrocatalysis and provide a novel strategy for developing non‐precious metal‐based electrocatalysts with broad energy conversion applications. Using EP and EA, we constructed P–N heterojunction MoSe_2_/PANI on NF, exhibiting high HER catalytic ability [36]. In single‐cell OER evaluations, MoSe_2_/PANI demonstrated comparable performance to commercial RuO_2_ in 1 m KOH, with a low overpotential of 1.61 V at 50 mA/cm^2^, a Tafel slope of 115.81 mV/dec, resistance of 2.5 Ω, and excellent long‐term stability, confirming that the P–N heterojunction enables bifunctional catalytic properties. However, limited by the theoretical water‐splitting potential (∼1.23 V vs RHE), the MoSe_2_/PANI system alone cannot achieve the high‐efficiency OER performance required for future applications. Introducing an effective cathode catalyst with excellent HER activity can balance the overall reaction and enhance OER performance at the anode. In dual‐electrode H‐cell tests, MoSe_2_/PANI served as both cathode and anode. Compared to Pt/C//MoSe_2_/PANI and Pt/C//RuO_2_ controls, MoSe_2_/PANI//MoSe_2_/PANI maintained stable HER performance at the cathode while significantly improving OER activity at the anode and overall stability. Notably, compared to the results obtained using the single cell, the Tafel slope of the anode decreased significantly from 115.81 to 65.17 mV/dec, and the resistance dropped from 2.5 Ω to ∼0.5 Ω, demonstrating that the MoSe_2_/PANI cathode effectively enhances anode performance. The system also exhibited an extremely low total overpotential between the cathode and anode of only 1.48 V at 10 mA/cm^2^ (1.67 V at 100 mA/cm^2^), with negligible changes after prolonged constant current density and continuous CV treatments, whereas Pt/C//RuO_2_ performance deteriorated significantly over time. Overall, this study reveals that the P–N heterojunction in MoSe_2_/PANI balances and optimizes water electrolysis, enhancing oxygen evolution at the anode and enabling high‐efficiency simultaneous hydrogen and oxygen production with low energy consumption. Therefore, with the rapid rise in demand for green energy, this emerging P‐N heterojunction concept has the potential to profoundly impact the development of emerging energy fields and related technologies, and provide strong technical support to achieve the goals of a low‐carbon economy and environmental protection.

## 4 Experimental Section

The experimental section, including the chemicals used in this study, experimental equipment, material synthesis and preparation, material analysis and characterization, and the electrochemical experiments, is provided in the Supplementary Information.

## Conflicts of Interest

The authors declare no conflicts of interest.

## Supporting information




**Supporting File**:advs75398‐sup‐0001‐SuppMat.docx.

**Supporting File**: advs75398‐sup‐0002‐VideoS1.mp4.

## Data Availability

Research data are not shared.
