# Supplementary material for: Unleashing the Power of P‐N Heterojunctions: Optimizing Water Electrolysis Efficiency Through a Cathode‐Driven Electrocatalyst Synergy
Source: Adv Sci (Weinh). 2026 Apr 20;13(40):e75398. doi: 10.1002/advs.75398 (PMC13335566; doi:10.1002/advs.75398)
Supplement: Supplementary file 1 — Supporting File:advs75398‐sup‐0001‐SuppMat.docx. [file ADVS-13-e75398-s001.docx]

**Supplementary Information**

Unleashing the Power of P-N Heterojunctions: Optimizing Water Electrolysis Efficiency Through a Cathode-Driven Electrocatalyst Synergy

Kumasser Kusse Kuchayita,^a^ Wei-Nien Su^a^ and Chih-Chia Cheng^ab^*

1. Graduate Institute of Applied Science and Technology, National Taiwan University of Science and Technology, Taipei 10607, Taiwan.

E-mail: [cccheng@mail.ntust.edu.tw](mailto:cccheng@mail.ntust.edu.tw)

1. Advanced Membrane Materials Research Center, National Taiwan University of Science and Technology, Taipei 10607, Taiwan.

* Corresponding author is marked with an asterisk (*) in the complete list of authors.

**Experimental Section**

**Materials**

The materials used in this study were of the highest available purity and were employed as received, unless otherwise specified. All chemicals and materials, including bulk molybdenum selenide crystals (MoSe_2_, 325 mesh, 99.9%), ruthenium oxide (RuO_2_, 99.5%), Nafion solution (5 wt. %), aniline (99.5%), potassium hydroxide (KOH, 85%), potassium chloride (KCl, 99%), the Ag/AgCl (3 M KCl) reference electrode, isopropanol (IPA, 99.8%), sulfuric acid (H_2_SO_4_, 97%), hydrochloric acid (HCl, 37%), platinum (Pt) wire, and graphite rod were sourced from Sigma-Aldrich (St. Louis, MO, USA) and/or Honeywell Fluka (Morristown, NJ, USA). Nickel foam (NF; > 99.96% purity, surface density of 346 g/m^2^, thickness 1.6 mm, porosity > 95%) was purchased from MTI Corporation (Richmond, CA, USA). Deionized (DI) water was used for all solution preparations. The 1.0 M KOH electrolytic solution, prepared in high-purity deionized water, had a pH of approximately 13.6. Water-soluble sodium-functionalized chitosan (Na-CMC) and Na-CMC-functionalized exfoliated MoSe_2_ nanosheets were prepared following the methods outlined in our previous publication.^[35]^

**Fabrication of MoSe_2_/PANI Electrocatalyst on NF by Electropolymerization (EP)**

The MoSe_2_/PANI electrocatalyst was prepared following our previously reported procedure.^[36]^ Briefly, composite working electrodes were fabricated by direct EP on NF using an electrochemical workstation (BioLogic SP-200, Seyssinet-Pariset, France) in a single-cell with a three-electrode array. In this setup, NF (1.0 cm × 1.0 cm, geometric area 1.0 cm^2^) served as the working electrode, Ag/AgCl (3 M KCl) was used as the reference electrode, and a Pt wire was the counter electrode. Prior to EP, the NF substrates were thoroughly cleaned to ensure good electrical contact and uniform film growth, to produce well-adhered and reproducible composite electrodes for subsequent electrochemical evaluation. The growth solution contained exfoliated MoSe_2_ nanosheets and saturated anilinium chloride (volume ratio 1:2). EP was performed galvanostatically at 0.1 mA/cm^2^ for 40 minutes, resulting in MoSe_2_/PANI on NF. After deposition, electrodes were rinsed thoroughly with DI water to remove loosely adhered nanosheets and residual salts, then air-dried at room temperature.

**Electroactivation (EA) of the Developed MoSe_2_/PANI Electrocatalyst on NF**

The EA process was performed following the procedure we previously established;^[36]^ in brief, the prepared working electrode was further electrochemically activated in 0.5 M H_2_SO_4_ solution by chronoamperometry (CP) at a constant current of 500 mA/cm^2^ for the optimal activation time. The EA step aims to enhance interfacial charge transfer, improve polymer doping, and strengthen the P–N heterojunction. After EA, the electrodes were rinsed with DI water and subsequently used for oxygen evolution reaction (OER) and overall water-splitting (OWS) evaluations in 1.0 M KOH.

**Fabrication of Pt/C/NF and RuO_2_/NF Benchmark Electrodes**

A homogeneous RuO_2_ catalyst ink was prepared by dispersing 5.0 mg of commercial RuO_2_ powder into 900 μL of a solvent mixture (isopropanol and water, 2:3 *v/v*) mixed with 100 μL of 5 wt.% Nafion solution. The suspension was sonicated in a water-bath ultrasonicator for 30 minutes to ensure homogeneity. To fabricate the RuO_2_/NF electrode, 250 μL of the catalyst ink was drop-cast onto a clean NF substrate (1 cm × 1 cm) and dried under ambient conditions. The same procedure was followed For Pt/C/NF, replacing commercial Pt/C for RuO_2_ in the ink formulation. Catalyst loadings were kept constant between the two electrodes.

**Physicochemical Characterizations**

The structural, morphological, and compositional characteristics of the composite nanomaterials (Na-CMC-functionalized exfoliated MoSe_2_ nanosheets) were analyzed using various techniques, including atomic force microscopy (AFM), dynamic light scattering (DLS), Raman spectroscopy, scanning electron microscopy (SEM), transmission electron microscopy (TEM), UV-visible (UV-Vis) spectroscopy, X-ray photoelectron spectroscopy (XPS), and X-ray diffraction (XRD), as described in our previous work.^[35]^

**Characterization of MoSe_2_/PANI on NF**

After the OER tests, the developed MoSe_2_/PANI electrode was characterized using Raman spectroscopy, XPS, and SEM, following previously reported methods.^[36]^

**Characterization of the Electrode after OER and OWS Testing**

Raman spectroscopy and SEM analyses were conducted both before and after the OER and OWS tests to assess the structural integrity of the electrode.

**Raman Spectroscopy:** Raman spectroscopy was performed on the NF substrate with deposited active components before and after OER testing. Prior to analysis, all NF samples were sectioned post-OER testing and dried under vacuum. Measurements were conducted using a Raman spectrometer (Jasco NRS-5100, Tokyo, Japan) with a spectral resolution of 1.09 cm^-1^ and a 20x objective lens at 25 °C, within the wavenumber range of 200‒500 cm^-1^. The excitation source was a 532 nm He-Ne laser.

**SEM:** The surface morphology and structural integrity of the NF were assessed using field-emission SEM (JSM-6500F, JEOL, Tokyo, Japan) before and after OER testing. Prior to imaging, the electrocatalyst-deposited NF samples were rinsed with distilled water, dried in a vacuum oven at 25 °C, and then sectioned into smaller pieces and coated with a thin layer of platinum via sputter coating to facilitate detailed morphological analysis. The elemental distribution across the samples was examined using energy-dispersive X-ray spectroscopy (EDX) integrated with NSS software, conducted at an accelerating voltage of 15.0 kV. To enable accurate elemental mapping and compositional analysis, the samples were fixed to an aluminum holder.

**Electrochemical Measurements (Single-Cell OER Testing)**

Electrochemical evaluations were conducted using an electrochemical workstation (BioLogic SP-200) in a conventional three-electrode setup (or single cell) at 25 °C with 1.0 M KOH as the electrolyte. The working electrode was the fabricated electrode with a geometric area of 1.0 cm^2^, the counter electrode was a carbon rod, and the reference electrode was an Ag/AgCl (3.0 M KCl) electrode. A Luggin capillary/salt bridge was employed to minimize uncompensated solution resistance between the reference and working electrodes. Prior to polarization measurements, the working electrode was conditioned by cyclic voltammetry (50 cycles) at a scan rate of 100 mV/s to stabilize the electrochemical interface. Linear sweep voltammetry (LSV) was subsequently carried out on the same workstation (BioLogic SP-200) at a scan rate of 5 mV/s to evaluate the OER performance of the catalyst.

**Potential Conversion and iR Compensation:** The potentials recorded with respect to the Ag/AgCl reference electrode were converted to the RHE scale using the following relation:

*E_RHE_ = E_Ag/AgCl_ + 0.059pH + E°_Ag/AgCl_*

where *E°_Ag/AgCl_* = 0.21 V at 25 °C for 3M KCl.

OER overpotential (η) was calculated as:

$$\eta= E_{RHE}-1.23 V$$

Furthermore, all potentials presented in the polarization curves were corrected for iR losses. The series resistance (R_s_) originating from the substrate and electrolyte was determined from the high-frequency intercepts in the EIS Nyquist plot and used for this compensation. The iR-corrected potential was determined using:

$$E_{corr}= E_{raw}-{iR}_{s}$$

where *i* is the measured current.

**Electrochemical Impedance Spectroscopy (EIS) Measurements:** EIS measurements were carried out using an electrochemical potentiostat (BioLogic SP-200) over a wide frequency range of 10^5^ to 0.1 Hz, applying a sinusoidal AC perturbation of 10 mV. The measurements were performed at a fixed overpotential of 300 mV vs. RHE to ensure a consistent electrochemical driving force across experiments and enable reliable comparison of catalytic activity under identical conditions. The resulting impedance spectra were fitted using a suitable equivalent circuit model to extract relevant electrochemical parameters.

**Overall Water Splitting (OWS) in the H-Cell (Full-Cell)**

**Nafion Membrane Activation and H-Cell Assembly**

Nafion 117 membranes (2.5 × 2.5 cm^2^, thickness 0.05 mm) were activated by sequential soaking in 3% H_2_O_2_, 0.5 M H_2_SO_4_, and DI water, with each step lasting 1 hour at 80 °C, followed by thorough rinsing to remove residual reagents. To evaluate OWS performance, a H-type electrochemical cell (Gaoss Union, Wuhan, China) was used, with the MoSe_2_/PANI bifunctional electrode acting as both the anode and cathode in a symmetric full-cell configuration. The cell was assembled using the commercial Nafion 117 membrane (DuPont, Fayetteville, NC, USA) and tested in 1.0 M KOH for overall water-splitting activity. A Luggin capillary connected to an Ag/AgCl (3 M KCl) reference electrode was used to minimize uncompensated resistance during diagnostic measurements.

**Durability and Accelerated Degradation Test**

Long-term stability was assessed by chronopotentiometry using an electrochemical workstation (BioLogic SP-200) at fixed current densities of 100 and 500 mA/cm^2^ for 24 h. The electrolyte was refreshed every 8 h to minimize performance drift due to concentration changes. Accelerated durability tests were also performed by subjecting the electrodes to 1000 cyclic voltammetry (CV) cycles at a scan rate of 100 mV/s within the potential range of 1.2–1.7 V vs. RHE. After these tests, the OER activity and relevant diagnostic parameters were reevaluated to assess performance degradation.

**Estimation of Electrochemically Active Surface Area (ECSA)**

The ECSA of the developed electrodes was estimated using CV to determine the electrochemical double-layer capacitance (*C*_dl_). CV measurements were conducted with a potentiostat (BioLogic SP-200) within the non-faradaic potential range of 1.18–1.28 V vs. RHE, at scan rates ranging from 20 to 160 mV/s. *C*_dl_ was calculated from the slope of the linear fit of half the current density difference, (J_a_−J_c_)/2, measured at 1.23 V vs. RHE, and plotted against the scan rate. The ECSA was then obtained by normalizing *C*_dl_ to the specific capacitance (*C*_s_), typically taken as 40 μF/cm^2^ for a smooth surface with an actual area of 1 cm^2^ in 1 M KOH using the following relations:

j_dl_ = ν *C*_dl_

ECSA = *C*_dl_/*C*_s_,

where j_dl_ is the double-layer charging current density and ν is the scan rate.

**Statistical Analysis**

All electrochemical measurements were performed in triplicate (at a minimum) using independently prepared electrodes. The reported values represent the mean, and representative curves along with fitted parameters were independently verified to ensure reproducibility.


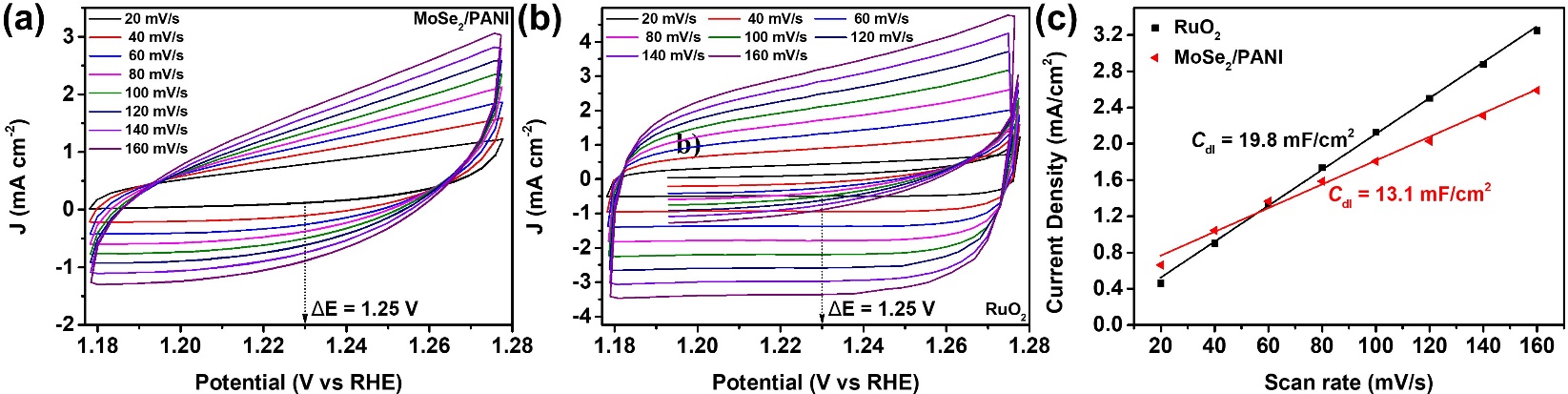


**Figure S1:** **(a)** CV curves for MoSe_2_/PANI and **(b)** RuO_2_ on NF at different scan rates in 1.0 M KOH aqueous solution. **(c)** Current density vs. scan rate plot and calculated *C*_dl_ values for MoSe_2_/PANI and RuO_2_ on NF in 1.0 M KOH solution.


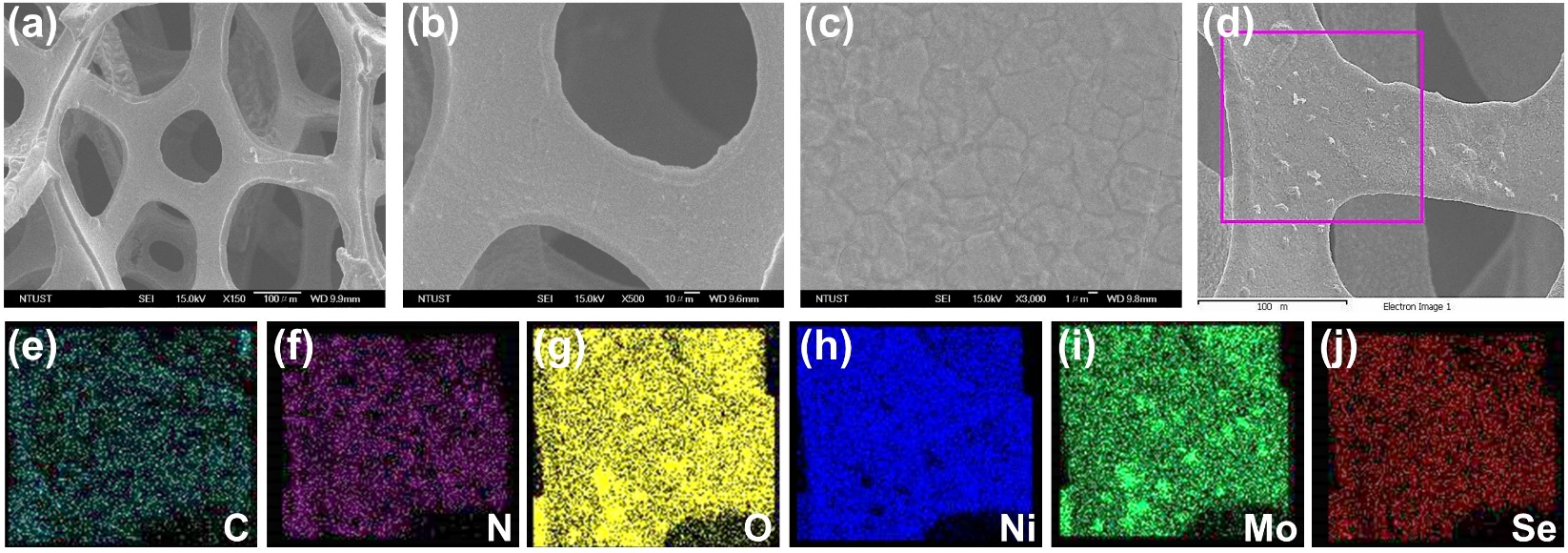


**Figure S2:** SEM images of MoSe_2_/PANI on NF after OER testing in a single cell with 1 M KOH solution, at magnifications of **(a)** ×150, **(b)** ×500, and **(c)** ×3000. **(d)** SEM image of MoSe_2_/PANI on NF after OER testing, used for elemental mapping analysis, with the analyzed region indicated by the purple box in the figure. Elemental mapping images corresponding to the purple box in (d): **(e)** C, **(f)** N, **(g)** O, **(h)** Ni, **(i)** Mo, and **(j)** Se.


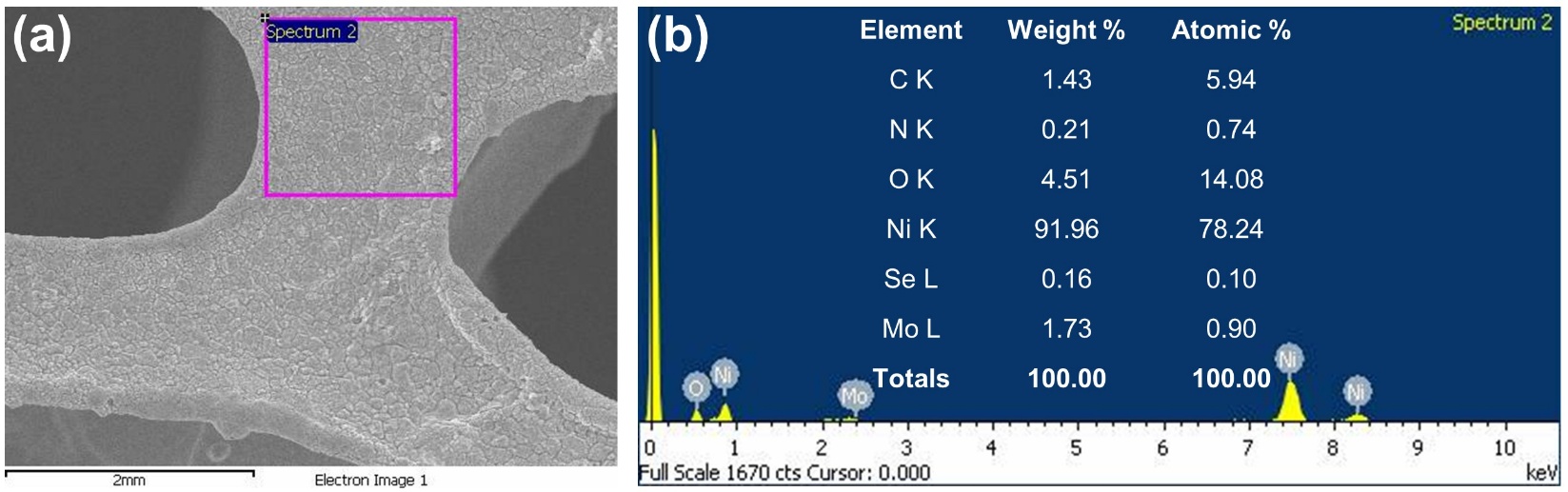


**Figure S3:** **(a)** SEM image of MoSe_2_/PANI on NF after OER testing in a single cell with 1 M KOH solution, used for EDX analysis, with the analyzed region indicated by the purple box. **(b)** Corresponding EDX spectrum for the region within the purple box in (a).


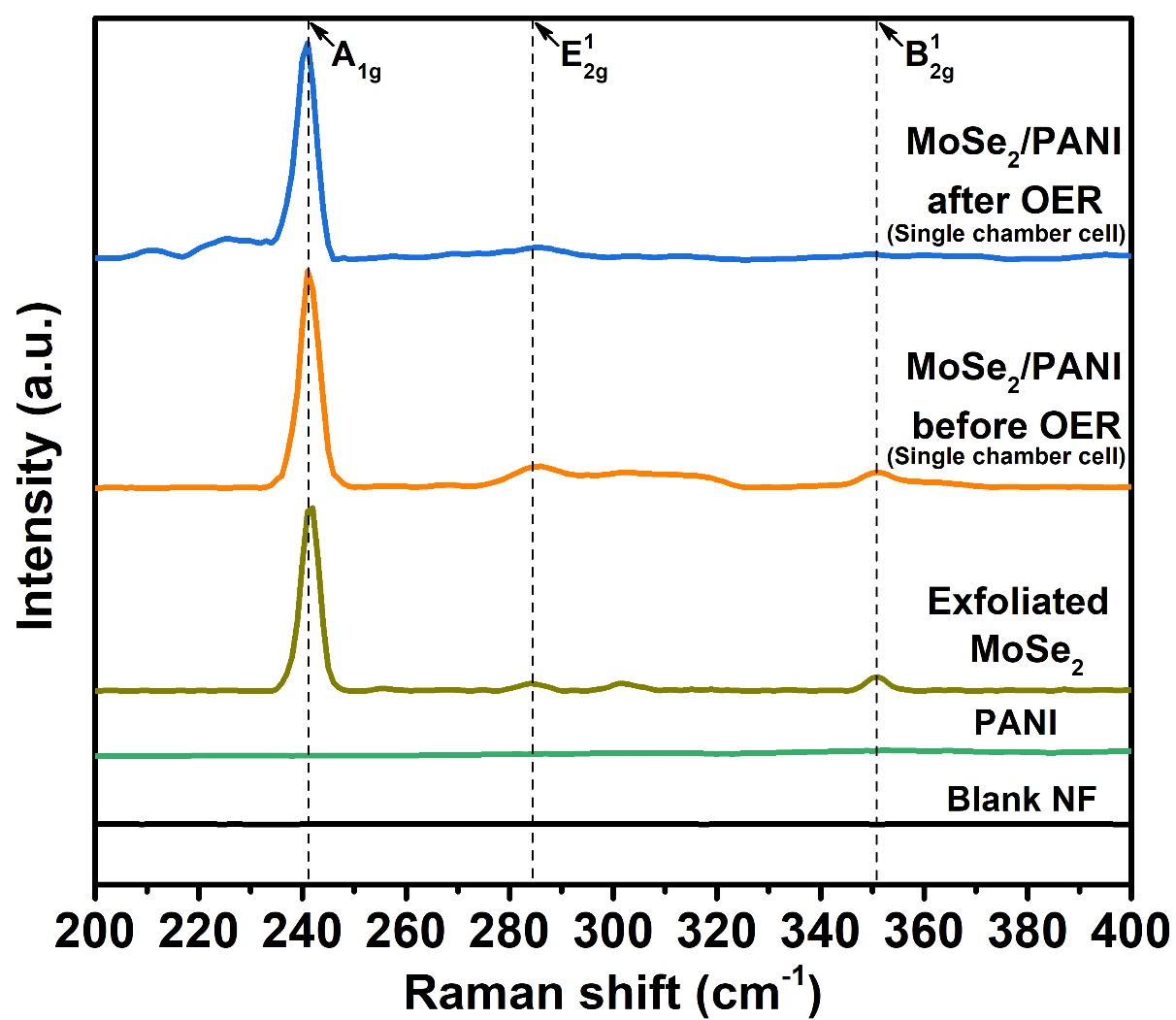


**Figure S4:** Raman spectra recorded at 25 °C for blank NF, pristine PANI, exfoliated MoSe_2_ nanosheets, and MoSe_2_/PANI on NF before and after OER testing in a single cell with 1 M KOH solution.


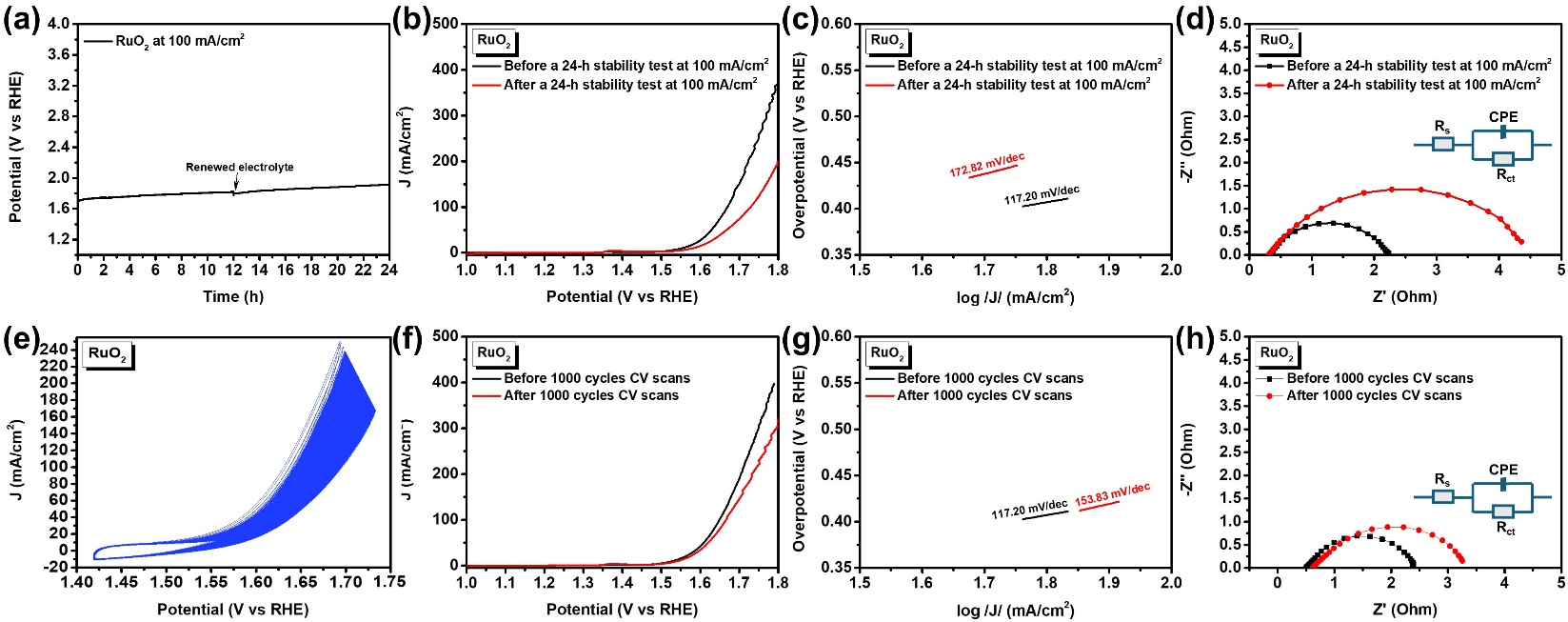


**Figure S5:** **(a)** Chronopotentiometry curve for the RuO_2_ electrocatalyst during continuous operation at a fixed current density of 100 mA/cm^2^ for 24 h in 1.0 M KOH solution. Electrochemical measurements for the RuO_2_ electrocatalyst in the single cell before and after 24 h of treatment at 100 mA/cm^2^ in 1.0 M KOH solution: **(b)** LSV curve, **(c)** Tafel plot, and **(d)** EIS spectra. **(e)** CV curve of RuO_2_ electrocatalyst in 1.0 M KOH solution after 1000 continuous cycles at a scan rate of 100 mV/s. Electrochemical measurements for the RuO_2_ electrocatalyst in the single cell before and after 1000 cycles of CV in 1.0 M KOH solution: **(f)** LSV curve, **(g)** Tafel plot, and **(h)** EIS spectra. Insets in (d) and (h) on the right show the equivalent circuit model used to fit the EIS data.


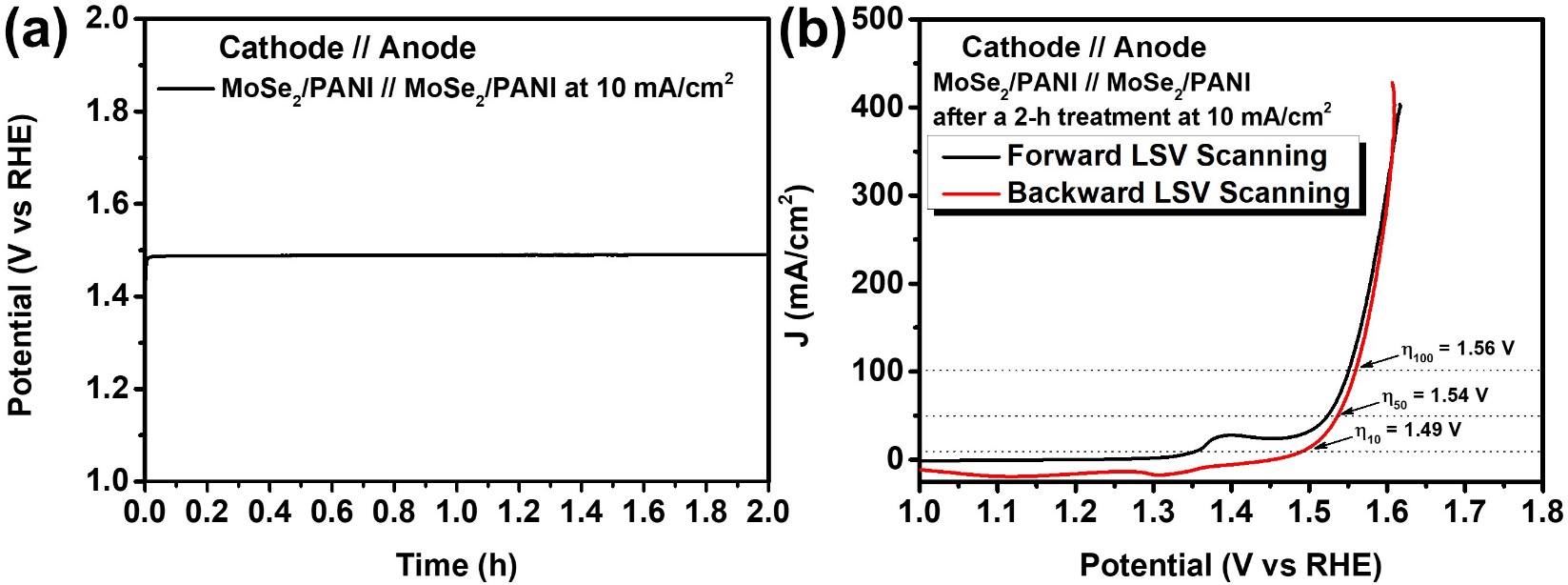


**Figure S6:** **(a)** Chronopotentiometry curve for MoSe_2_/PANI on NF during continuous operation at 10 mA/cm^2^ for 2 h in 1.0 M KOH. **(b)** Forward (black) and backward (red) LSV curves for MoSe_2_/PANI on NF in the H-cell after 2 h at 10 mA/cm^2^ in 1.0 M KOH.


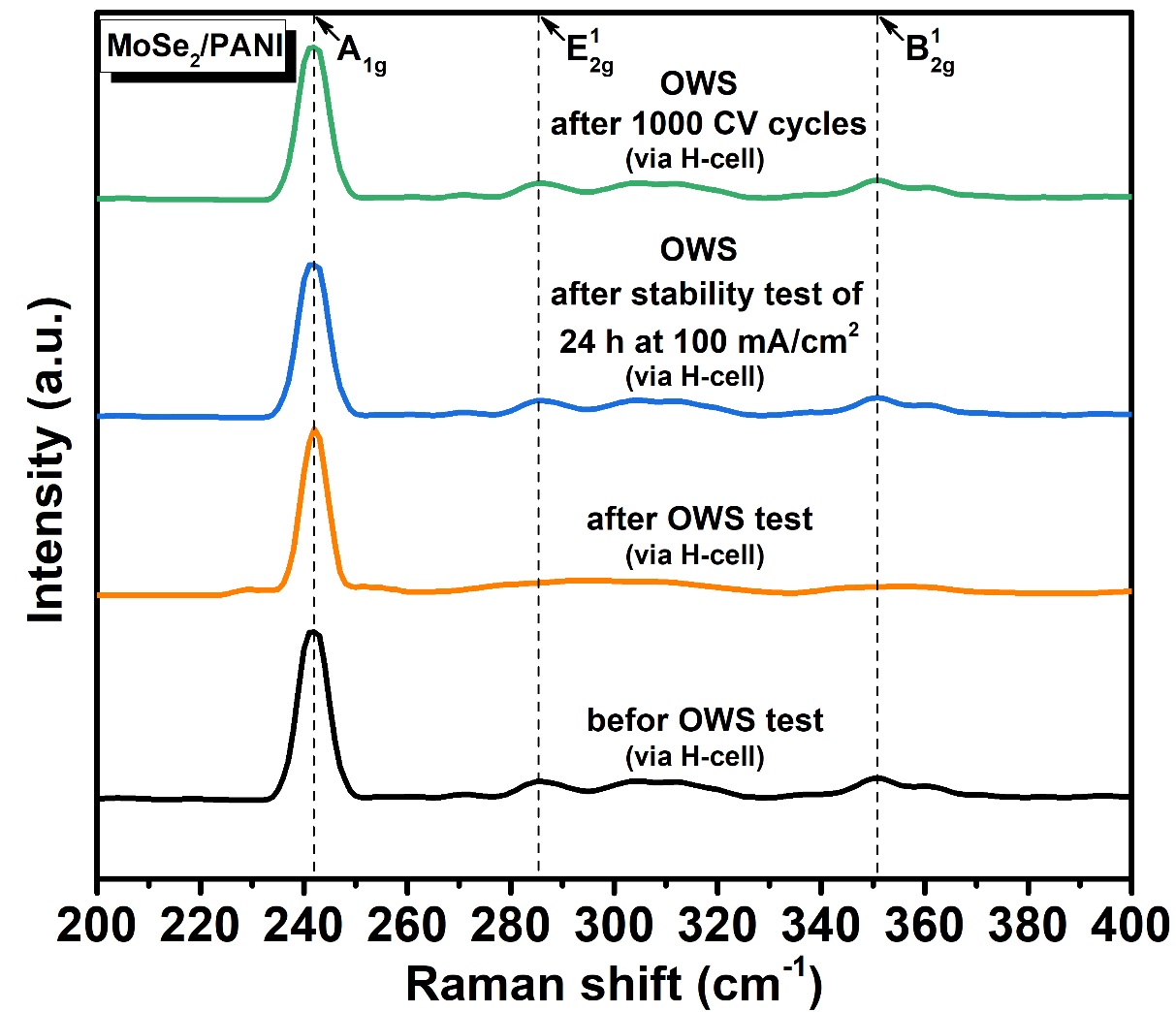


**Figure S7:** Raman spectra recorded at 25 °C for MoSe_2_/PANI/NF after 1000 cycles at a scan rate of 100 mV/s or continuous operation at a fixed current density of 100 mA/cm^2^ for 24 h in 1.0 M KOH, followed by overall water splitting (OWS) testing in the H-cell.

**Table S1:** Comparison of the overall water splitting performance of MoSe_2_/PANI on NF with recently reported bifunctional electrocatalysts.

| **No** | **Catalyst** | **Electrolyte** | **Total overpotential (V) at 10 mA/cm^2^** | **Reference** |
| --- | --- | --- | --- | --- |
| 1 | 10%Co-MoSe_2_@PANI100 on GCE | 1 M KOH | 1.82 | (33) |
| 2 | Ni(OH)_2_/Ni_3_S_2_ | 1M KOH | 1.49 | (52) |
| 3 | CNT@NiSe/SS-400 | 1M KOH | 1.71 | (53) |
| 4 | NiSe/NF | 1 M KOH | 1.69 | (54) |
| 5 | NiSe_2_/Ni | 1 M KOH | 1.64 | (55) |
| 6 | CoFe/NF | 1 M KOH | 1.64 | (56) |
| 7 | Sand rose-like MoSe_2_/NiSe/NiFe‑LDH | 1 M KOH | 1.51 | (57) |
| 8 | Straw-like MoSe_2_–NiFeSe/NF | 1 M KOH | 1.54 | (58) |
| 9 | CoS_2_/GF // CoS_2_/GF | 1 M KOH | 1.74 at 20 mA/cm^2^ | (59) |
| 10 | MoSe_2_/PANI | 1 M KOH | 1.48 | This work |
| 11 | Pt/C // RuO_2_ | 1 M KOH | 1.55 | This work |

**Video S1:** MoSe_2_/PANI on NF demonstrating OER catalytic performance in a single cell with 1.0 M KOH aqueous solution.
